# Inflammatory Alteration of Human T Cells Exposed Continuously to Asbestos

**DOI:** 10.3390/ijms19020504

**Published:** 2018-02-08

**Authors:** Naoko Kumagai-Takei, Shoko Yamamoto, Suni Lee, Megumi Maeda, Hidenori Masuzzaki, Nagisa Sada, Min Yu, Kei Yoshitome, Yasumitsu Nishimura, Takemi Otsuki

**Affiliations:** 1 Department of Hygiene, Kawasaki Medical School, 577 Matsushima, Kurashiki, Okayama 701-0192, Japan; kumagai@med.kawasaki-m.ac.jp (N.K.-T.); s.yamamoto@med.kawasaki-m.ac.jp (S.Y.); slee@med.kawasaki-m.ac.jp (S.L.); nagisada@okayama-u.ac.jp (N.S.); yumin06@hotmail.com (M.Y.); kei_y@med.kawasaki-m.ac.jp (K.Y.); yas@med.kawasaki-m.ac.jp (Y.N.); 2 Department of Biofunctional Chemistry, Division of Bioscience, Okayama University Graduate School of Natural Science and Technology, 1-1-1 Tsushima-Naka, Kita-Ku, Okayama 700-8530, Japan; mmaeda@okayama-u.ac.jp; 3 Department of Life Science, Faculty of Life and Environmental Science, Prefectural University of Hiroshima, Shobara, Hiroshima 727-0023, Japan; hmatsuzaki@pu-hiroshima.ac.jp; 4 Department of Biophysical Chemistry, Graduate School of Medicine, Dentistry and Pharmaceutical Sciences, Okayama University, Okayama 700-8530, Japan; 5 Department of Occupational and Environmental Health Science, School of Public Health, Peking University, 38 Xueyuan Road, Beijing 100191, China; 6 Department of Occupational Diseases, Zhejiang, Academy of Medical Sciences, 182 Tian Mu Shan Road, Zhejiang 310013, China

**Keywords:** asbestos, T cell, apoptosis, inflammation, cell surface molecule, IL-6, interferon-γ

## Abstract

Asbestos is a known carcinogen and exposure can lead to lung cancer and malignant mesothelioma. To examine the effects of asbestos fibers on human immune cells, the human T cell leukemia/lymphoma virus (HTLV)-1 immortalized human T cell line MT-2 was employed. Following continuous exposure to asbestos fibers for more than eight months, MT-2 sublines showed acquisition of resistance to asbestos-induced apoptosis with decreased death signals and increased surviving signals. These sublines showed various characteristics that suggested a reduction in anti-tumor immunity. On the other hand, inflammatory changes such as expression of MMP7, CXCR5, CXCL13 and CD44 was found to be markedly higher in sublines continuously exposed to asbestos compared with original MT-2 cells. All of these molecules contribute to lung inflammation, T and B cell interactions and connections between mesothelial cells and T cells. Thus, further investigation focusing on these molecules may shed light on the role of chronic inflammation caused by asbestos exposure and the occurrence of malignant mesothelioma. Finally, regarding peripheral T cells from healthy donors (HD) and asbestos-exposed patients with pleural plaque (PP) or malignant pleural mesothelioma (MPM), following stimulation of CD4+ T cells, T cells from MPM patients showed reduced potential of interferon (IFN)-γ expression. Moreover, levels of interleukin (IL)-6, one of the most important cytokines in chronic inflammation, in cultured supernatants were higher in PP and MPM patients compared with HD. Overall, asbestos-induced chronic inflammation in the lung as well as the pleural cavity may facilitate the onset of asbestos-induced cancers due to alterations in the interactions among fibers, immune cells such as T and B cells and macrophages, and mesothelial and lung epithelial cells. Further investigations regarding chronic inflammation caused by asbestos fibers may assist in identifying molecular targets for preventive and therapeutic strategies related to the effects of asbestos exposure.

## 1. Introduction

Asbestos is known to cause various benign and malignant pulmonary diseases [1,2]. Non-cancerous disease such as asbestosis is defined as progressive pulmonary fibrosis with many patients suffering from shortness of breath, coughing, wheezing and chest pain [3,4]. Since the fibrosis is progressive, asbestosis causes impairment of the quality of life, which is often lifelong even though it is non-cancerous. Additionally, pleural benign pathological conditions such as diffuse pleural thickening, pleural effusion and pleural plaque (PP) are found in people exposed to asbestos [5]. These also result in some respiratory symptoms, although PP is usually indicative of asbestos exposure and there are no physical disturbances in patients with PP. These individuals are only diagnosed following radiological findings [3,4,5].

Additionally, asbestos causes malignant diseases such as lung cancer and malignant mesothelioma (MPM) [6,7,8,9]. Furthermore, the International Agency for Research on Cancer (IARC) has classified asbestos as a carcinogenic agent with sufficient evidence in humans for cancers of the larynx and ovary as well as lung and cases of MPM [10].

Considering the carcinogenic activity of asbestos fibers, the dominant mechanism at play involves the production of reactive oxygen species (ROS) as well as reactive nitrogen species (RNS) due to iron contained in the fibers, particularly in crocidolite and amosite [11,12,13,14]. Through the Fenton reaction, the produced ROS and RNS cause DNA damage in surrounding cells. Additionally, alveolar macrophages (AMs), which act on foreign substances via phagocytosis, are unable to digest asbestos fibers because of the fiber length and rigidity. Thereafter, AMs fall into a so-called frustrated state and produce ROS [15,16]. Thus, ROS somehow overflow in the pulmonary area and gradually transform cells located near asbestos fibers towards a cancerous state with concomitant accumulation of DNA damage in these cells [11,12,13,14]. Moreover, cells surrounding asbestos fibers sometimes tend to incorporate the fibers which may physically damage the chromosomes. On the other hand, asbestos fibers remaining in the pulmonary area adsorb various inhaled carcinogenic substances derived from tobacco smoke, air pollution and other factors [16,17]. Depending on the nature of these various mechanisms at play, asbestos fibers can cause malignant transformation.

As asbestos fibers initially enter the human body, the role of the inflammasome located in antigen-presenting cells (APC) such as AMs is important in the recognition of outer (asbestos fibers, silica particles and nanomaterials) as well as inner (crystals formed by cholesterols and uric acids) danger signals [18,19,20]. The inflammasome comprises caspase 1, apoptosis-associated speck-like protein containing a CARD (ASC), nucleotide-binding oligomerization domain (NOD)-like receptors (NLRs) and caspase 11, and promotes the maturation of inflammatory cytokines such as interleukin (IL)-1β and IL-18 [18,19,20]. Regarding asbestos-induced fibrosis and cancers, the slow and continuous progression of inflammations caused by asbestos-exposed APC/AMs is important because of long-term persistency of inhaled fibers in the pulmonary region and the occurrence of fibrosis and cancers in the long-term following initial exposure to asbestos in asbestosis patients as well as in patients with asbestos-induced cancers.

When considering the chronic inflammation caused by asbestos exposure, the cellular alterations in T cells are also important since the interaction between T cells and APC/AMs may influence progression of the continuous inflammation. Thus, the cellular and molecular modifications in human T cells caused by continuous and relatively low-dose exposure to asbestos is introduced in this review from the viewpoint of chronic inflammation.

## 2. Establishment of a Human T Cell Model for Continuous and Low-Dose Exposure to Asbestos Fibers

Many reports have appeared concerning investigations using alveolar epithelial cells and pleural mesothelial cells to explore cellular changes induced by transient and relatively high-dose exposure to asbestos fibers [21,22,23,24,25]. These cells produced ROS and RNS and proceeded into apoptosis via activation of the mitochondrial apoptotic pathway. Then, it was considered that the accumulation of DNA damage as well as the acquisition of resistance against apoptosis are the cellular mechanisms of malignant transformation [21,22,23,24,25].

What about immune cells? In our initial study, a variety of cell types were exposed to asbestos fibers such as human T and B cell-derived cell lines including tumor cells from T or B cell leukemias and lymphomas, myelomas, as well as virus-immortalized cell lines such as Epstein-Barr virus immortalized lymphoblastoid cell lines (EBVLCLs) and the human T cell leukemia/lymphoma virus (HTLV)-1 immortalized T cell line MT-2 [26]. Among these cells, tumor-derived cell lines were resistant to asbestos-induced growth inhibition, however, EBVLCLs and MT-2 cells were sensitive at the same doses. Thereafter, MT-2 was chosen (T cells may be important in exploring immune alterations caused by asbestos fibers) for further investigations [26].

The cellular and molecular mechanisms at play in MT-2 cells following transient and relatively high-dose (causing growth inhibition) exposure to asbestos fibers (chrysotile A was used in this initial investigation since it was the major form of asbestos fiber used in the industrial field) were initially examined. MT-2 cells showed production of ROS, activation/phosphorylation of pro-apoptotic molecules involved in the mitogen-activated protein kinase (MAPK) pathway such as p38 and JUN, release of cytochrome-c from mitochondria to cytoplasm, an increase in the BAX/Bcl-2 ratio, and activation of caspase-9 and -3 towards apoptosis [27]. Following this initial investigation, crocidolite exposure was also examined and similar cellular changes were observed [28].

**Figure 1:**
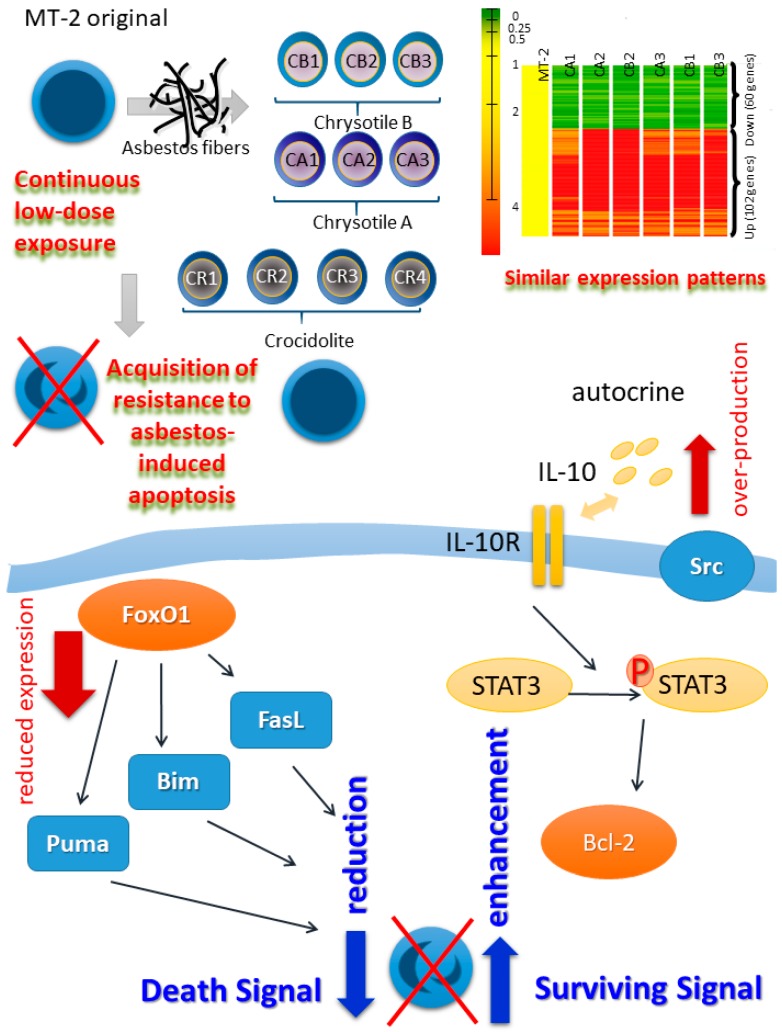
The effects of continuous and relatively low-dose exposure of asbestos fibers chrysotile A and B, and crocidolite were investigated using the human T cell leukemia/lymphoma virus (HTLV)-1 immortalized MT-2 polyclonal T cell line. Following more than eight months of continuous exposure, all sublines (CB1–3, CA1–3 and CR1–4) showed acquisition of resistance to asbestos-induced apoptosis. Additionally, cDNA microarray assays revealed a similar pattern in all sublines. Mechanistic analyses of the apoptosis resistance showed decreased death signals and increased surviving signals. Reduced expression of FoxO1 transcription factor resulted in a reduction in levels of pro-apoptotic molecules such as *Puma*, *Bim* and *Fas ligand* (FasL) from the decreased death signal. Overproduction of (interleukin) IL-10, phosphorylation of STAT3, and up-regulation of Bcl-2 resulted in increased surviving signals in sublines continuously exposed to asbestos.

Thereafter, continuous and relatively low-dose exposure (the doses of asbestos utilized were determined from those doses which induced less than half of the MT-2 cells to proceed towards apoptosis following transient exposure) of the MT-2 cell line was initiated and continued for more than eight months, with monitoring of the appearance of apoptosis by monthly high-dose exposure to asbestos [29]. After eight months, the continuously exposed sub-line of MT-2 showed acquisition of resistance to asbestos-induced apoptosis [29]. As shown in Figure 1, independently established sublines, three for chrysotile B, three for chrysotile A and four for crocidolite, were established and all sublines acquired resistance to asbestos-induced apoptosis [28]. Comparison of cDNA microarray assays of the six sublines continuously exposed to chrysotile A and B with the original MT-2 line revealed similar patterns in all sublines regarding up-regulated and down-regulated genes, indicating that investigations of the cellular and molecular alterations in these sublines may confirm the effects of continuous exposure to asbestos on T cells [30,31]. Crocidolite-exposed sublines also showed similar characteristics with respect to cytokine production profiles and resistance to apoptosis via changes in the BAX/Bcl-2 ratio [28].

Following further investigations of the cellular and molecular modifications involved in the acquisition of resistance to asbestos-induced apoptosis, dual mechanistic changes were found as shown in Figure 1. One of these changes represents a decrease in the death signal. All continuously exposed sublines revealed drastic reductions in the expression of forkhead box protein O1 (FoxO1), a transcription factor, in terms of both mRNA and protein levels [32]. Thereafter, the expression of certain FoxO1-regulated pro-apoptotic molecules such as *puma*, *bim* and *fas ligand* decreased in sublines continuously exposed to asbestos. Additionally, molecular transient knock-down of FoxO1 in original MT-2 cells resulted in a reduction in the occurrence of apoptosis, and forced expression of FoxO1 in the CA1 subline caused a re-increase in expression of the *puma* gene [32]. These results indicated that death signals in continuously exposed sublines decreased following asbestos exposure [32]. The other change represents an increase in surviving signals. All of the sublines showed increased secretion of IL-10 [29]. Since MT-2 cells possess IL-10 receptors on their surface, these were utilized in the autocrine mechanism. Thereafter, the signal transducer and activator of transcription 3 (STAT3) was phosphorylated. Since Bcl-2 is known to be located downstream of STAT3, Bcl-2 was up-regulated in all sublines. The importance of overexpressed Bcl-2 was confirmed by transient silencing of Bcl-2 in the CB1 subline, causing enhancement of apoptosis and reduced growth inhibition following transient asbestos exposure [29].

Additional information was derived from various findings concerning the reduction of anti-tumor immunity using these sublines. Reduced expression of C-X-C motif chemokine receptor 3 (CXCR3) was found in sublines as well as ex vivo*-*activated freshly isolated human peripheral T cells with asbestos fibers [30,31]. A reduction in CXCR3 expression was also found in peripheral CD4 positive T cells derived from PP and MPM patients. Since CXCR3 is important in summoning tumor-attacking T cells in tumor-surrounding areas, a reduction in CXCR3 expression in T cells indicates weakened anti-tumor immunity [30,31]. Additionally, since MT-2 cells possess regulatory T cell (Treg)-like function, the inhibitory activity of continuously exposed MT-2 sublines was compared with that of original MT-2 cells [33]. The study revealed enhancement of Treg function in sublines by cell-cell contact as well as enhanced production of Treg-soluble factors such as IL-10 and transforming growth factor (TGF)-β. Additionally, it is known that FoxO1 also regulates cell cycle-related genes to suppress progression of the cell cycle [34]. Then, since reduced expression of FoxO1 was found in all sublines, cell cycle progression was accelerated in sublines compared with MT-2 original cells with increased expression of cyclins, marked expression of cyclin D1, and reduction in cyclin-dependent kinases-inhibitors (CDK-Is) such as p21Cip1, p57Kip2 and others [34]. These findings indicated that asbestos-exposed Treg cells may have enhanced function and proliferation resulting in a reduction in anti-tumor immunity [33,34].

## 3. Alterations in Inflammation-Related Molecules in MT-2 Sublines Continuously Exposed to Asbestos

**Figure 2:**
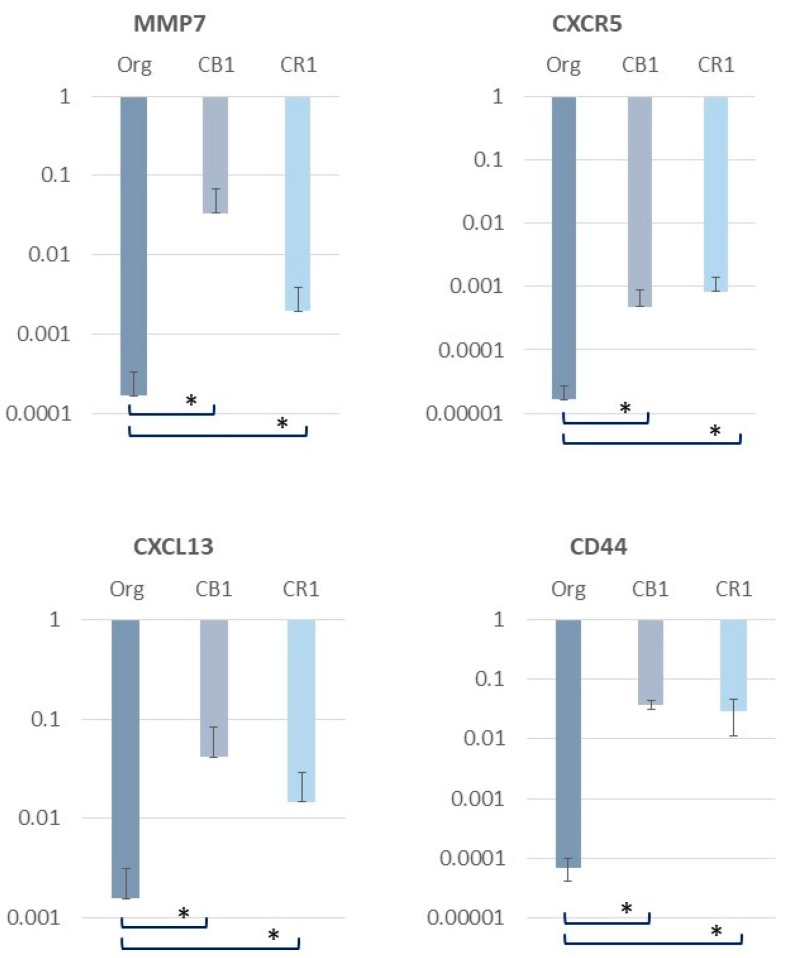
Real-time RT-PCR revealed significantly higher expression levels of MMP7, CXCR5, CXCL13 and CD44 in a subline of MT-2 continuously exposed to asbestos, CB1 exposed to chrysotile B and CR1 exposed to crocidolite, compared with the expression in original MT-2 cells untreated with asbestos fibers. Statistical significance was analyzed using Log_10_ values, student *t* test,* *p* < 0.05.

As a result of the cDNA micro array data (performed twice), genes related to inflammatory changes in T cells were identified and re-examined using real-time reverse transcriptase polymerase chain reaction (RT-PCR) methods. As shown in Figure 2, several interesting genes were up-regulated in MT-2 sublines continuously exposed to asbestos (results using CB1 and CR1 sublines are shown). Although the results shown in Figure 2 are yet to be published, these findings are representative of the microarray data. Thus, further experimental investigations in addition to studies using specimens derived from asbestos-exposed patients will be needed. However, presenting these preliminary findings here may support the findings of altered inflammatory responses caused by long-term and continuous exposure to asbestos.

Matrix metalloproteinase-7 (MMP-7), a member of the MMP family and the smallest of the known MMPs, was up-regulated. MMP7 was reported to be necessary in cases of lung injury to recruit neutrophils and facilitate re-epithelialization by syndecan-1 shedding [35]. Additionally, MMP7 is expressed in epithelial cells and is considered as a biomarker of parenchymal lung diseases such as pulmonary fibrosis [36,37]. Furthermore, increased expression was found in hyperplastic lung epithelial cells. If T cells continuously exposed to asbestos fibers produce MMP7 persistently, MMP7 may be involved in the occurrence of recurrent lung epithelial injury caused by asbestos fibers and remodeling processes in the lung. The repeating injury and remodeling may result in some escape from normal healing mechanisms and induce an aberrant proliferating scenario in pulmonary epithelial cells that facilitates the development of lung cancer. Although it is unclear whether these events are present in pleural mesothelial cells, MMP7 may be important in terms of asbestos-induced fibrosis as well as cancer-related changes in cells.

CXCR5 expression also increased in sublines continuously exposed to asbestos. CXCR5, also known as CD185, plays a role in facilitating the migration of T cells to the lymph node B cell zone [38,39,40]. The role of CXCR5-expressing T cells on B follicle homing receptor and its roles in inflammation and immune reactions remains unclear. However, if CXCR5 is up-regulated in T cells continuously exposed to asbestos, certain communicating interactions between T and B cells may occur and be persistently enhanced in regional lymph nodes. This situation indicates that continuous inflammatory alterations occur in asbestos-related lymph nodes that cause lung as well as pleural inflammation for prolonged periods.

The chemokine (C-X-C motif) ligand 13 (CXCL13), also known as B lymphocyte chemoattractant (BLC) or B cell-attracting chemokine 1 (BCA-1), is also up-regulated in the MT-2 subline exposed continuously to asbestos. Similar to the aforementioned case of CXCR5, CXCL13 is also known to play a role in the interaction between T and B cells [41,42,43,44]. Additionally, CXCL13 is considered to play an important role in the formation of tertiary lymphoid follicles in the respiratory tract in response to infections and chronic inflammations. From the viewpoint of the long-lasting effects of asbestos remaining in the pulmonary spaces and the persistent interactions among asbestos fibers and immune cells, increased expression of CXCL13 may contribute to the formation of chronic inflammation in the lung and the occurrence of fibrosis, as well as cancerous changes in lung epithelial and mesothelial cells.

Finally, CD44 is up-regulated in MT-2 sublines continuously exposed to asbestos. CD44 is known as a receptor for hyaluronic acid, which is a biomarker of asbestos-induced mesothelioma in pleural effusion [45,46,47,48]. Additionally, CD44 interacts with osteopontin, which is another biomarker of mesothelioma, certain collagens, and MMPs. Thus, the increased expression of CD44 in asbestos-exposed T cells may be important with respect to the interaction between inflammatory and mesothelial cells from the viewpoint of immune cell-editing malignant transformation as well as tumor cell-editing alterations in immune cells.

As previously mentioned, MT-2 sublines continuously exposed to asbestos showed up-regulation of various molecules important for lung inflammation, interactions with B cells, and formation of immune inflammatory reactions as well as cell-cell communication between T and mesothelial cells. Further investigations of these up-regulated molecules in asbestos-exposed cells and the occurrence of asbestos-causing cancers should be performed to identify potential preventive or therapeutic target molecules in asbestos-related diseases as well as cancers.

## 4. Alterations in Cytokine Production in Peripheral T Cells Derived from Asbestos-Exposed Patients

**Figure 3:**
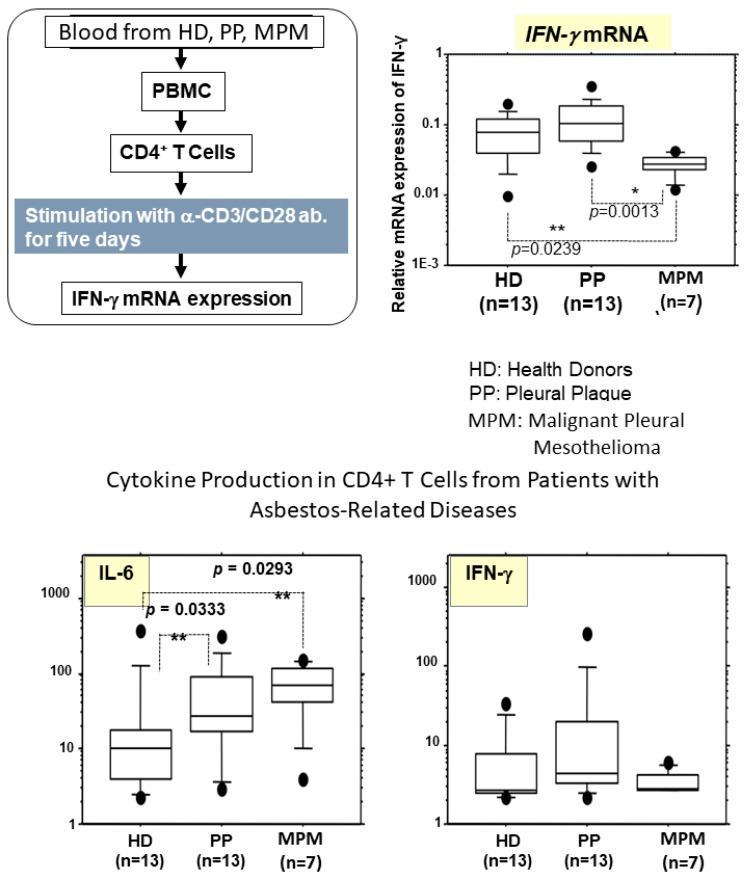
To investigate T cell functions altered by asbestos exposure, freshly isolated peripheral CD4+ T cells from healthy donors (HD), patients with pleural plaque (PP) or malignant mesothelioma (MPM) were stimulated with anti-CD3/28 antibodies for five days and IFN-γ mRNA expression was monitored. As a result, it was found that IFN-γ expression in CD4+ T cells from MPM patients was lower compared with that in cells derived from HD and PP. Regarding cytokines produced during stimulation of CD4+ T cells derived from HD, PP and MPM, IL-6 levels were higher in culture supernatants from PP and MPM compared with that from HD, although IFN-γ levels did not differ in supernatants from HD, PP and MPM. The *p*-value was obtained using the Mann-Whitney *U* test. * *p* < 0.01, ** *p* < 0.01. PBMC: peripheral blood mononuclear cells.

As previously reported [30,31] and mentioned above, expression of CXCR3 in CD4+ T cells derived from peripheral blood of asbestos-exposed patients with PP or MPM was reduced. These findings showed reduced expression of CXCR3 in MT-2 sublines as well as freshly isolated peripheral CD4+ T cells derived from healthy volunteers and cultured ex vivo with asbestos fibers [30,31]. As shown in Figure 3, during these experiments using MT-2 and MT-2 sublines, cDNA array and pathway analyses using array data indicated that the interferon-γ pathway was down-regulated in sublines continuously exposed to asbestos [30,31]. Thus, in the experiments using peripheral blood CD4+ T cells activated with anti-CD3 and CD28 for five days, IFN-γ mRNA expression was assayed. As a result, it was found that expression of IFN-γ mRNA was significantly lower in CD4+ T cells derived from MPM patients compared with those cells from healthy donors (HD) or PP patients. IFN-γ is known to play an important role in attacking tumors via T cells and other immune cells, where asbestos exposure and the occurrence of asbestos-induced cancer may have an effect on T cells and reduce anti-tumor immunity. Additionally, with the aforementioned ex vivo stimulation of T cells derived from HD, and PP and MPM patients, cytokine production was assayed. Examination of IFN-γ expression did not show any significant differences, although IFN-γ mRNA expression was reduced in CD4+ T cells derived from MPM patients [30,31]. However, one interesting finding was the observed increased expression of IL-6 in asbestos-exposed patients, PP and MPM patients compared with HD. Actually, our previous data that measured cytokines in the serum of HD, and PP and MPM patients revealed a significant increase in IL-6 expression only in the serum of MPM patients [49]. These findings suggest that asbestos exposure causes chronic inflammation presented by an increase in the IL-6-producing potency of CD4+ T cells, although the occurrence of mesothelioma may alter the inflammatory profiles as revealed by the reduced IFN-γ-producing activity in MPM patients and the absence of significant increases in serum IL-6 levels in PP patients [30,31]. Further investigations will be necessary to delineate the precise mechanisms involved in asbestos-induced inflammation and the observed cytokine profiles, and these efforts may eventually lead to the identification of potential target molecules that can be utilized for the prevention as well therapy of asbestos-induced mesothelioma.

## 5. Conclusions

In this brief review, the effects of continuous asbestos exposure on human T cells using the MT-2 cell line as well as findings using peripheral blood CD4+ T cells were introduced from the viewpoint of inflammation. Asbestos-induced cancers such as lung cancer and mesothelioma may be derived from chronic inflammation caused by asbestos exposure and altered interactions among various immune cells such as macrophages, T cells including effector T cells, regulatory T and Th17 cells, B cells, and others. Additionally, mesothelial and lung epithelial cells may also play important roles in asbestos-induced chronic inflammations via interaction with immune cells. From our findings, MMP7, CXCR5, CXCL13 and CD44 expression was up-regulated in MT-2 sublines continuously exposed to asbestos, and suggested interactions with these epithelial and mesothelial cells.

We have been investigating anti-tumor immunity caused by chronic exposure of various immune cells to asbestos such as natural killer (NK) cells [50,51] and cytotoxic T lymphocytes (CTLs) [52,53,54], in addition to Treg [33,34] and effector T [30,31] cells, and have found that asbestos exposure induced a reduction in anti-tumor immunity. To delineate the tumor-promoting mechanisms induced by exposure to asbestos fibers, the investigation of immune and target cells such as mesothelial or lung epithelial cells may lead to the identification of certain molecular targets that play significant roles in asbestos-induced chronic inflammation as well as carcinogenesis.

Additionally, given the various alterations found in our cell-line model and clinical specimens, it may be possible to generate a formula to detect asbestos exposure as well as the occurrence of MPM, particularly in groups at high risk of asbestos exposure such as present and past workers handling asbestos, building demolition contractors, workers in rubble processing companies, and present and past residences near asbestos-handling factories. To date, the radiological diagnostic method employed to identify PP or asbestos-induced alterations is the only method that is used to screen for asbestos exposure and the occurrence of MPM. However, if a formula can be developed that utilizes immunological modifications such as increases in IL-10, TGF-β, changes in cell surface markers in immune cells such as CXCR3 in CD4+ cells and NKp46 in NK cells, this would assist in the initial line screening to avoid unnecessary radiation exposure and to reduce the expense and man-power associated with the initial screening.

Moreover, our findings may contribute towards the development of novel immunological therapies, especially with respect to immune-checkpoint medications for MPM. Since asbestos exposure alters immune cell functions, from the viewpoint of anti-tumor immunity, the effectiveness of standard immune check point inhibitors may be reduced in asbestos exposed patients. Thus, it will be necessary to bind the ideas of immune alterations induced by asbestos exposure and explore the newer candidates for immune check point inhibitors.

## Abbreviations

PP	pleural plaque
MPM	malignant pleural mesothelioma
IARC	International Agency for Research on Cancer
ROS	reactive oxygen species
RNS	reactive nitrogen species
AMs	alveolar macrophages
APC	antigen-presenting cells
ASC	apoptosis-associated speck-like protein containing a CARD
NLRs	nucleotide-binding oligomerization domain-like receptors (NOD-like receptors)
IL	interleukin
EBVLCLs	Epstein-Barr virus immortalized lymphoblastoid cell lines
HTLV	human T cell leukemia/lymphoma virus
MAPK	mitogen-activated protein kinase
FoxO1	forkhead box protein O1
STAT3	signal transducer and activator of transcription 3
CXCR3	C-X-C motif chemokine receptor 3
Treg	regulatory T cell
TGF	transforming growth factor
CDK-Is	cyclin-dependent kinases-inhibitors
MMP-7	Matrix metalloproteinase-7
CXCL13	chemokine (C-X-C motif) ligand 13
BLC	B lymphocyte chemoattractant
BCA-1	B cell-attracting chemokine 1
IFN	interferon
HD	healthy donors
NK	natural killer
CTLs	cytotoxic T lymphocytes
